# Milk Improvement

**Published:** 1920-02-07

**Authors:** 


### Milk Improvement.

### Mr. Wilfred Buckley and Grading.

NO ONE questions the vital importance of supplying a greater quantity and a better quality of milk in this country. It is, however, a very complicated problem, and one is glad that it is being tackled by so enthusiastic an advocate of a large and wholesome milk supply as Mr. Wilfred Buckley, the Director of Milk Supplies at the Ministry of Food. Mr. Buckley has great faith in the new grading plans for milk, which he believes will result in an improvement in its hygienic quality.

In a paper on the question which he read to the Glasgow and West of Scotland Agricultural Discussion Circle, Mr. Buckley stated that we consumed too little milk and produced too little during the winter period—an opinion he emphasised with the statement that if all the children in the country up to fourteen years of age were to have the full supplies that they ideally required for their proper maintenance and growth there would be no milk left for any other purpose during the months of November, December, January, and February.

Examining the effect of grading, he said that the production of Grade A milk, and in particular of Grade A (certified) milk, which should be milk of the highest hygienic quality that it was possible to produce, irrespective of cost, would not only provide a supply for those who were in a financial position to buy it, but it would have a far more important result, inasmuch as it would set up a standard and thus be the means not only of educating the public as to what milk should be, and could be, but of teaching all concerned in its production and distribution—farm hands, farmers, and distributors—how milk should be produced and handled under the most careful conditions. The better grades of milk would command a higher price at the farm; therefore a direct financial incentive would be given to the farmer to produce cleaner and more wholesome milk. The progressive dealer who was willing and anxious to sell on a genuine and honest basis would be aided in his efforts. It would no longer be possible for the dishonest dealer to sell the lowest grade of milk as "nursery" or "invalids'" milk. The public would be enabled to purchase the quality of milk they required and for which they could afford to pay. If by means of grading or by any other means a supply of really clean, wholesome milk were created it would not be confined to the use of the rich. Hospitals would be enabled to supply their patients with wholesome milk, it would reach many children of the poorest classes through infant welfare centres, and many of the public who were not rich would find the means to buy for their children milk that they knew to be safe. The inevitable result of grading would be to raise the hygienic standard of the lowest grade of milk, and in this way all classes would benefit.

Mr. Buckley laid particular emphasis on the need for improving the conditions under which milk was produced—the sterilisation by steam of every kind of utensil, cleaner sheds, clean cows, and the immediate cooling of the milk and its maintenance, at as low a temperature as possible.

